# Influence of Pyrolysis Temperature on Physico-Chemical Properties of Corn Stover (*Zea mays* L.) Biochar and Feasibility for Carbon Capture and Energy Balance

**DOI:** 10.1371/journal.pone.0156894

**Published:** 2016-06-21

**Authors:** Muhammad Khalid Rafiq, Robert Thomas Bachmann, Muhammad Tariq Rafiq, Zhanhuan Shang, Stephen Joseph, Ruijun Long

**Affiliations:** 1 College of Pastoral Agriculture, Science and Technology Agric, Lanzhou University, 222 Tianshui South Road, Lanzhou.730000, PR China; 2 Directorate of Range Mgt and Forestry, Pakistan Agricultural Research Council, Islamabad, 44000, Pakistan; 3 Malaysian Institute for Chemical and Bioengineering Technology (MICET), Universiti Kuala Lumpur (UniKL), Lot 1988, Taboh Naning,78000 Alor Gajah, Melaka, Malaysia; 4 Department of Environmental Science, International Islamic University, Islamabad, 44000 Pakistan; 5 State Key Laboratory of Grassland Agro-ecosystems, International Centre for Tibetan Plateau Ecosystem Management, School of Life Sciences, Lanzhou University, Lanzhou, 730000 China; 6 School of Materials Science and Engineering, University of New South Wales, Sydney, NSW 2052, Australia; University of Akron, UNITED STATES

## Abstract

This study examined the influence of pyrolysis temperature on biochar characteristics and evaluated its suitability for carbon capture and energy production. Biochar was produced from corn stover using slow pyrolysis at 300, 400 and 500°C and 2 hrs holding time. The experimental biochars were characterized by elemental analysis, BET, FTIR, TGA/DTA, NMR (C-13). Higher heating value (HHV) of feedstock and biochars was measured using bomb calorimeter. Results show that carbon content of corn stover biochar increased from 45.5% to 64.5%, with increasing pyrolysis temperatures. A decrease in H:C and O:C ratios as well as volatile matter, coupled with increase in the concentration of aromatic carbon in the biochar as determined by FTIR and NMR (C-13) demonstrates a higher biochar carbon stability at 500°C. It was estimated that corn stover pyrolysed at 500°C could provide of 10.12 MJ/kg thermal energy. Pyrolysis is therefore a potential technology with its carbon-negative, energy positive and soil amendment benefits thus creating win- win scenario.

## 1. Introduction

Sustainable economic development, food security and environmental management are some of the highest priorities of the modern world as these are concerned with the present and future generations. The multidimensional crises of global climate change, energy and water shortages as well as agricultural land degradation due to nutrient depletion provide major social, political, and economic challenges of today’s world. [1]

China is the second highest emitter of CO2. In December 2009, China’s State Council declared that the country aims to reduce its 2005 carbon emissions by 40 to 45% in year 2020. If successful, this would have a considerable positive impact not only for China but also the rest of the world. [2–3] China has been an agricultural country for millennia, and tens of millions of people are still involved in the agriculture sector which provides both and income and food security. [4] Some of the main commodities produced include rice paddy, vegetables, tomato, apples, wheat, potato and corn accounting for 18 to 59% of the world’s production (Table 1). [5]

**Table 1:** Main agricultural commodities produced in China.

Agriculture commodity	China [Mt/yr]	World [Mt/yr]	%
Vegetables	160000000	269852343	59%
Apples	37000000	76378738	48%
Tomatoes	50000000	161793834	31%
Paddy	204236000	738187642	28%
Cotton	6840000	25955096	26%
Potatoes	87260000	365365367	24%
Corn	205614000	872791597	24%
Wheat	121023000	671496872	18%

Clare et al (2015) reported that there is over 800 million tonnes of agricultural crop straw that China produces each year, up to 40% of which is burned in-field as a waste.[6] Residues from agricultural produce are therefore an abundant and renewable energy source with potentially low net CO_2_ emission. Corn is one of the main crops cultivated in China and production accounts for 24% in the world (Table 1). [5] According to China Statistical Yearbook (National Bureau of Statistics of China, 2013) [7], 0.2 billion tons of corn was produced and around 0.4 billion tons of corn stover (CS) was generated in 2012. In addition, a 100% increase in corn production has been observed in China over the past 20 years. Compared to rice, wheat, potatoes and cotton, corn belongs to C_4_ plants characterized by a higher yield potential, lower erosion-index, better CO_2_ reduction rates and need for less fertilizer, water and chemicals.[8]

At present, more than 70% of CS are land filled or burnt due to the high cost of collection, transportation, and low price paid to the farmers for their residue.[9] Even though illegal in most parts of China many farmers with burn their crop residues emits significant quantities of green house gases into air like CO_2_, N_2_O, CH_4_, organic compounds (VOCs) as well as semi-volatile organic compounds (SVOCs) and other particulates.[10] Therefore, it is necessary to develop environmental friendly and effective technologies to utilize agricultural residues such as CS in order to alleviate the environmental and energy issues.[11] Modern thermo-chemical energy conversion technologies such as combustion, gasification and pyrolysis provide clean energy from waste biomass and facilitate, to varying degrees, climate change mitigation.[4] Pyrolysis, the heating of biomass in the absence or very limited presence of oxygen to produce primarily biochar but also bio-oil and some pyrogas, is attractive because of its carbon negative property.[12]. Biochar can be used as an effective amendment in degraded or low fertility soils with substantial environmental benefits [13]. Life cycle assessment of biochars produced from corn stover, yard waste and switchgrass revealed that the net greenhouse gas emissions for both corn stover and yard waste were negative, at -864 and -885 kg CO_2_ equivalent emissions reductions per tonne dry feedstock, respectively. Of these total reductions, 62–66% is realized from C sequestration in the biochar.[14]

Few studies have partially investigated the effect of pyrolysis temperature on biochar yield [15,16,17] elemental composition [15,16,17,18] ash content [15,16,17,18] and composition [15,16] volatile matter [15,16,17] pH [15,16] cation exchange capacity [15,16] BET (N_2_) [15,17,18] and electrical conductivity [4,15]. Quantitative knowledge of biochar-relevant parameters is important to assess the quality and commercial value of the biochar as well as the potential suitability, stability and impact of the biochar on the fertility of a given soil.[19] In addition, empirical mathematical relationships between process conditions such as pyrolysis temperature and physico-chemical properties of biochar enable researcher and practitioner alike to select suitable process conditions to produce biochar of desired properties with minimum trial and error.

The objectives of this research, therefore, are to independently produce and characterize biochar from corn stover at different pyrolysis temperatures, consolidate the available literature on biochar production from corn stover for comparison purposes as well as to establish common trends and empirical relationships, identify and discuss potential reasons for variations and, last but not least, highlight gaps in knowledge for future research. In addition this study will look into associated soil amendment; energy and carbon capture potential of the experimental biochar.

## 2. Material and Methods

### 2.1. Ethics Statement

The corn stover used in this study was collected from agricultural fields around Lanzhou. No specific permissions were required for the described locations because corn stover was the left over waste having no use for the farmers. We confirm that the field studies did not involve endangered or protected species.

### 2.2. Biochar production

Corn stover was collected from agricultural fields (104^°^09’E, 35^°^56’N, altitude 1750 m) around Lanzhou, China and the biochar production method was used as described by Uzoma et al.[20]. In brief, the dried feedstock was cut into 1–2 cm long pieces, placed on stainless steel trays, covered with a tightly fitting lid, and pyrolyzed under oxygen limited conditions in a muffle furnace. The CS was heated at a rate of 20°C min^−1^ to a final temperature of 300°C, 400°C or 500°C respectively, and held for 2 hours. All biochar samples were ground and sieved at <0.154 mm for their physical and chemical analyses. Experiments were carried out in triplicate.

### 2.3. Yield and elemental analysis of biochars

The experimental biochar samples were tested for ash, pH, total Ca, Mg, K, Na, total phosphorus (P), carbon (C), hydrogen (H), nitrogen (N), oxygen (O). Yield percentage of biochar was determined by following equation:

Yield (%) = (weight of biochar) / (weight of feedstock) ×100 %.

Ash content was analyzed by heating biochar samples at 500°C for 8 h in a muffle furnace [21]. The relative ash content was then calculated as follows:

Ash (%) = (weight of ash)/(weight of biochar)×100 %.

The pH of biochar was measured in deionized water at the ratio of 1:5 wt/wt with a calibrated pH meter.[21] The elemental composition was determined according to Enders et al. (2012)[16] using an elemental analyzer from Elementar Analysensysteme GmbH (varioELcube). Nutrient elements Ca, K, Mg, Na, and P were measured using an inductively coupled plasma-atomic emission spectrometer (IRIS ER/S). Before analysis, the biochar sample (about 0.05 g) was first digested by the concentrated HNO_3_/H_2_O_2_ solutions.[21]

### 2.4. Surface and Physical Characterization

The determination of BET surface area, crystal structures, functional groups and pyrolytic performance and thermal resistance were carried out in the analysis laboratory of College of Chemistry and Chemical Engineering, Lanzhou University, PR China. The Brunauer–Emmett–Teller (BET) surface area y N_2_ gas sorption analysis at 77 K in a relative pressure from 0.05 to 0.35 using a Nova 2200e surface area analyzer (Tristar3200, Micromeritics, USA) after degassing at -195°C for a minimum of 8 h. The total pore volume of the biochars was estimated to be the liquid adsorbate volume of N_2_ adsorbed at a relative pressure of about 0.99. The average pore width was calculated as follows: [22]

average pore width= (4× (total pore volume value)/(BET value)

The surface functional groups of the biochars were determined by Fourier Transform Infrared (FTIR) spectroscopy using a Nicolet 380 spectrophotometer (Avatar370, Thermo Nicolet, USA). Addition of solid KBr to the biochars provided dilution and homogenization. The spectra were performed at 4 cm^−1^ resolution and a mirror velocity of 0.48 cm S^−1^ with a 400 to 4,000-cm^−1^ scan range. X-ray diffraction (XRD) was used to observe the changes in mineral crystals between the biochars, using a computer controlled diffractometer (X'Pert PRO). The scans were collected from 0–60° using Cu–Ka radiation (40 kV, 40 mA) at a scan rate of 1° min^−1^. The significant phase peaks were tentatively identified by comparing the XRD patterns with the mineral database in the resulting biochars. Thermo-gravimetric analysis (TGA) was performed on a Q-5000IR (TA Instruments, USA) coupled with a differential thermal analyzer (DTA). For the TGA experiment, 5 mg of each sample was processed and heated up to 700°C at a heating rate of 10°C / min. Nitrogen was used as a carrier gas and applied at a flow rate of 25 ml/min. The ^13^C nuclear magnetic resonance (NMR) spectrum of the 500°C biochar were obtained on an AVANCE 400WB (Bruker, Germany) using the cross-polarization magic angle spinning (CP/MAS) technique.

### 2.5. Energy Measurements of Corn Stover and Biochars

Bomb calorimeter was used to estimate higher heating value (HHV) of corn stover feedstock and biochars prepared at 400 and 500°C. Moisture content of corn stover and biochars was determined by drying at 60°C to constant weight. Hydrogen % of the corn stover and biochars was determined by using Elementar Analysensysteme GmbH (varioELcube). Following equation was used to measure the lower heating value (LHV) of the corn feedstock and biochars.[23]

LHV = HHV – 2.454 × (W + 9H)

Where HHV = higher heating value [MJ/kg], W = moisture content [%-wt] on dry basis and H = hydrogen content [%-wt.] The maximum net potential energy available during the pyrolysis was estimated using the following equation. [24]

Maximum energy from pyrolysis = biomass HHV – (char HHV × char yield)

## 3. Statistical analysis

A SPSS (version 18.0) statistical software programme was used to perform statistical analysis of the data for physico-chemical properties of experimental biochars and energy values. Significant differences between treatments were based on pyrolysis temperatures using one way analysis of variance (ANOVA). Means were separated by least significant difference (LSD) test, at 5% level.

## 4. Results and Discussion

### 4.1. Biochar Yield and Chemical Characteristics of Biochars

Biochar yield from corn stover and its chemical properties are summarized in Table 2. The yield of biochar decreased as pyrolysis temperature increased. The physico-chemical and structural characteristics of biochar were significantly influenced by pyrolysis temperature (p≤0.05).

**Table 2:** Yield and Physico-Chemical Properties of Biochars. Mean values followed by different letters within same rows are significantly different at P ≤ 0.05.

Parameter	Pyrolysis Temperature		
	300°C	400°C	500°C
Yield [%]	66±2 a	37±1 b	29.2±0.3 c
pH [H_2_O]	7.70±0.08 c	8.8±0.2 b	9.775±0.005 a
Ash [%]	5.7±0.2 c	12.5±0.2 b	18.7±0.3 a
Volatile matter (%)	54.0±0.6 a	45.5±0.5b	33.8±0.5 c
C [%]	45.5±0.5 b	64±1 a	64.5± 1 a
H [%]	5.4±0.6 a	3.9±0.3 b	2.7±0.1 c
O [%]	42±1 a	32±1 b	33.1±0.4 b
N [%]	0.63±0.04 a	0.42±0.04 b	0.25±0.01 c
P [mg L^-1^]	3.4±0.1 c	7.2±0.3 a	5.54±0.10 b
K [mg L^-1^]	60.1±0.2 c	102± 2 b	224±2 a
Ca [mg L^-1^]	21.0±0.3 c	24.1±0.2 b	29.7±0.1 a
Mg [mg L^-1^]	18.8±0.6 c	22.6±0.7 b	30.3±0.4 a
Na [mg L^-1^]	1.78±0.02 b	2.03±0.04 b	3.3±0.4 a
Total Cation Base [mg L^-1^]	101.7±0.5 c	151±2 b	288±2 a

**Fig 1:**
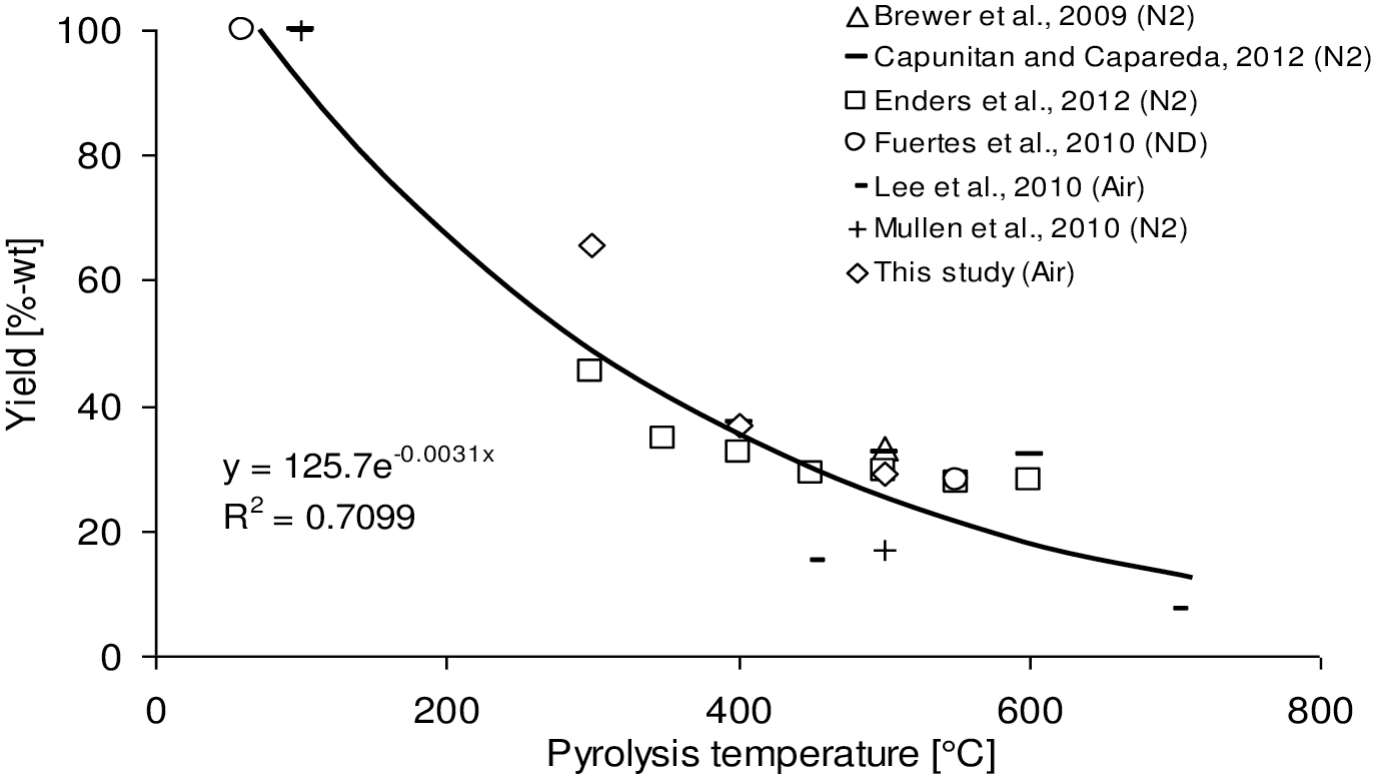
Effect of pyrolysis temperature on biochar yield from corn stover produced at different heating rates, holding times, particle sizes, reactor atmosphere and type (n = 22).

The degree of carbonization for biochar was accelerated with increasing pyrolysis temperature from 300°C, 400°C to 500°C. When plotting the biochar yields at different pyrolysis temperatures and comparing with literature, a general exponential decrease can be observed (Fig 1). [16,17,25,26,27,28]

The primary thermal degradation of biomass happens during pyrolysis. The pyrolytic volatiles are further broken into low molecular weight organics and gases as the pyrolysis temperature increases. [29] The results are also in agreement with the findings of the TGA (section 4.5). In TGA study of biochars the weight loss is maximum at 300°C suggesting more volatile matters compared to chars produced at 400°C and 500°C.

**Fig 2:**
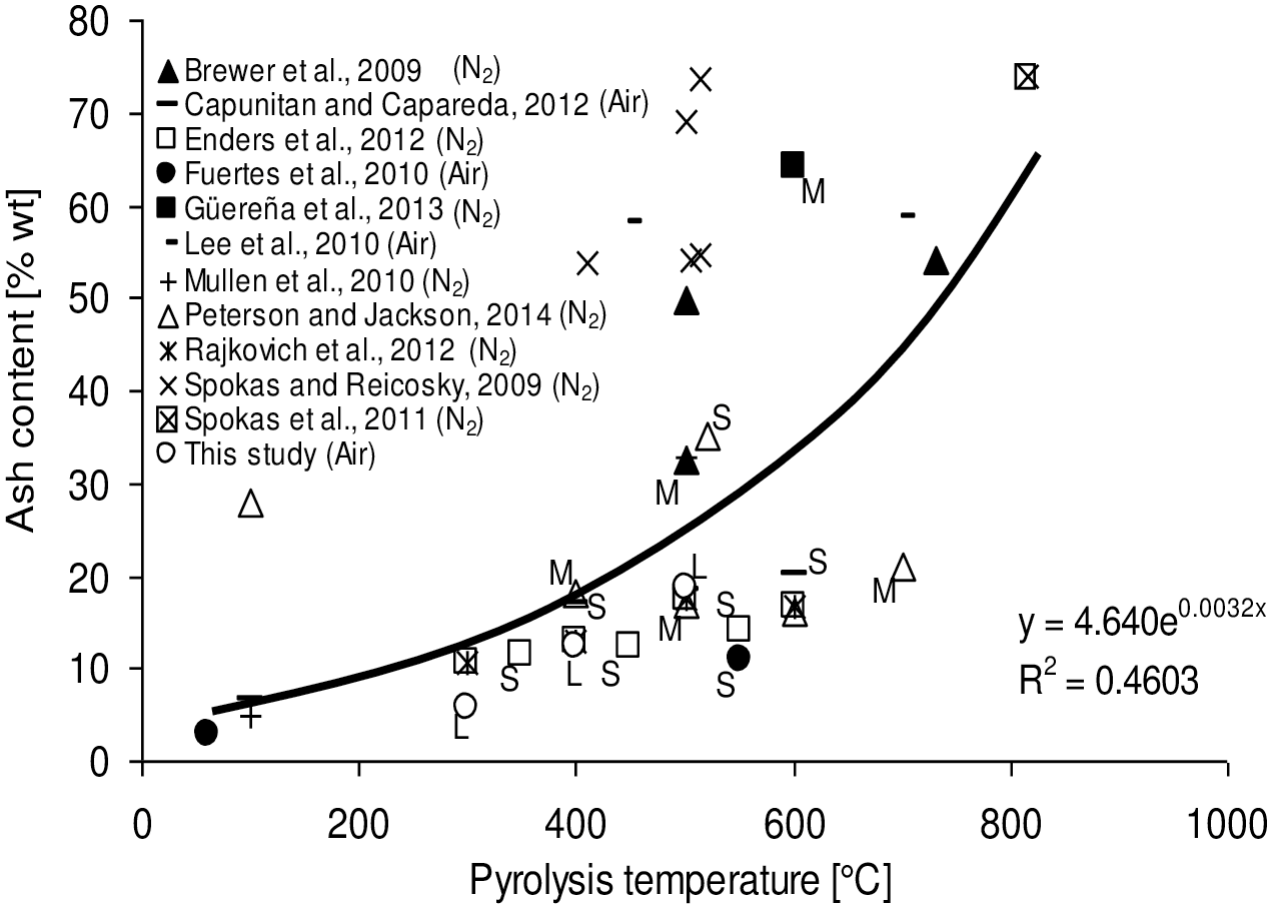
Effect of pyrolysis temperature on ash content in biochar from corn stover (n = 41). Where available, pyrolysis holding time of a given data point is indicated by a letter with S—short (<0.5 hrs), M–medium (0.5–1 hr) and L–long (>1 hr). Atmosphere present inside the pyrolysis reactor is stated after the reference.

The relative ash content of the resulting biochars increased with increasing temperature up to 500°C. The increase in ash content from 300–500°C is the result of a progressive concentration of minerals and destructive volatilization of ligno-cellulosic matters as temperature increased.[30,31] Comparing the findings with related literature also reveals an exponential increase of ash content with pyrolysis temperature (Fig 2). [15,16,17,18,25,26,27,28,32,33,34]

**Fig 3:**
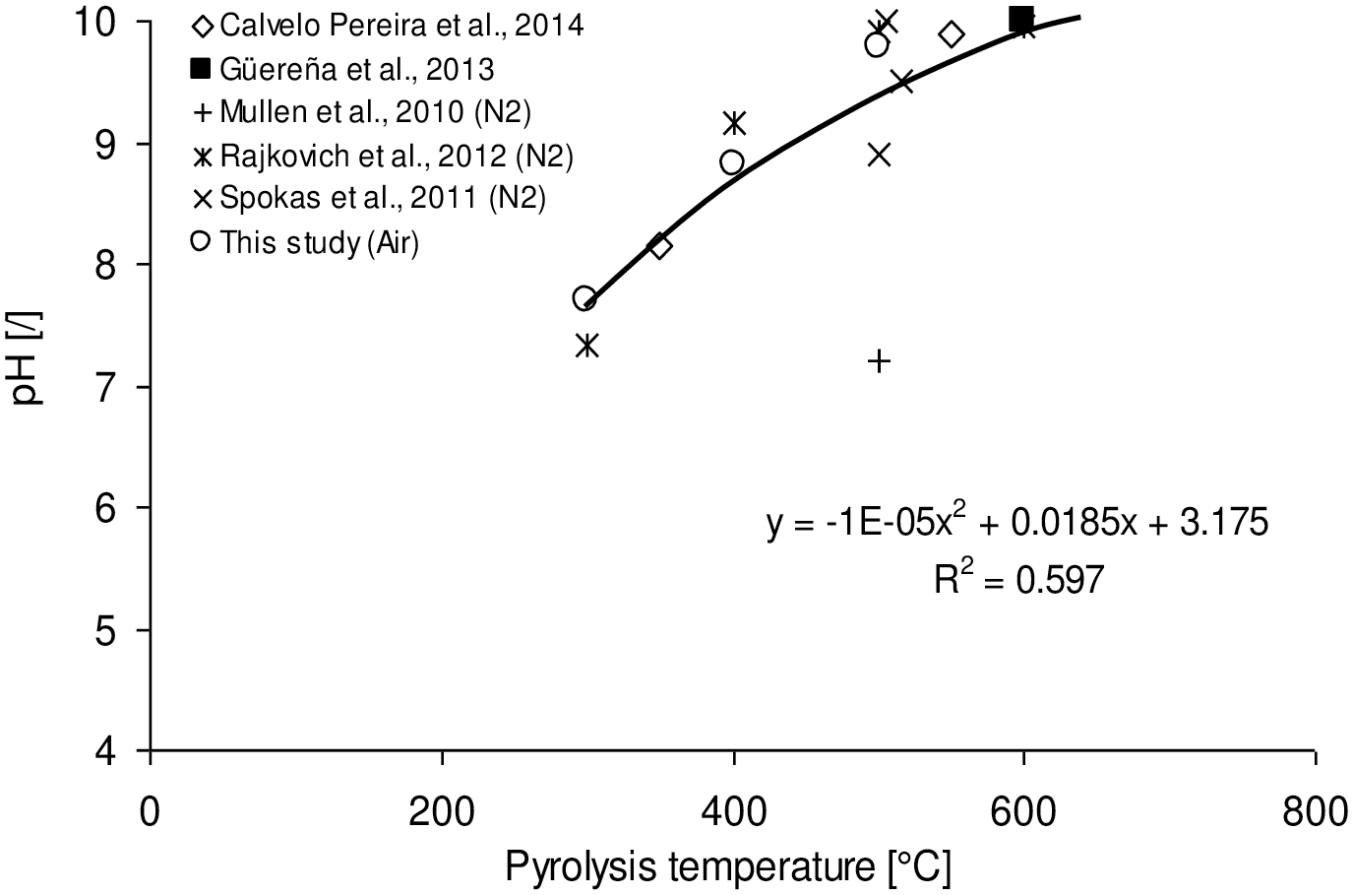
Effect of pyrolysis temperature on pH of biochar produced from corn stover (n = 15). All pH values were determined in distilled or deionized water but at different char to water ratios.

However, the correlation coefficient of 0.46 is comparatively low partially due to inclusion of apparent outliers. For example, one study reported an ash content in original corn stover of 28% (wt) [18] which is 4–10 times higher than any value reported elsewhere.[17,26,35,36,37] The high ash contents in CS char reported are probably due to presence of foreign material since no effort was made to control for the dirt content of the stover.[38] In addition the methods used to determine ash content varied widely with ASTM D1762-84 (Standard Test Method for Chemical Analysis of Wood Charcoal) used by four groups, ASTM D7582 (Proximate Analysis of Coal and Coke by Macro Thermogravimetric Analysis) used by three, ASTM D3172 (Standard Practice for Proximate Analysis of Coal and Coke) by two, ASTM E870-82 (Standard Test Methods for Analysis of Wood Fuels) used by one, while in-house methods (e.g. heating at 650°C for 6 h) and external labs where also deployed in three cases. It has been suggested that some methodological limitations of the ASTM D1762 method exist which can cause the loss of certain inorganic elements such as P, K, S and fractions of carbonates at temperatures as low as 500°C thus resulting in a negative bias in reported ash values.[16] The use of air or inert gas N_2_, different holding times and heating rates are not able to individually explain why some biochars had greater ash contents than others at comparable temperatures. Other factors such as representativeness of temperature measurements especially in upscaled reactors, representative sampling, sample storage conditions (e.g. duration, temperature, atmosphere) and number of replicates used for proximate analysis (Enders et al., 2012 duplicate; Brewer triplicate; all other studies considered here did not state number of replicates and did not report standard deviations[16,25] are expected to affect the determination of ash content and should be taken into account in the experimental design, execution and discussion of results. It is also suggested to initiate a study which determines the measurement uncertainty in ash content analysis as is required for ISO 17025 accredited laboratories. Determining and reporting accurate ash contents is important because this parameter is frequently used in elemental analysis to estimate the oxygen content in chars by difference [16’17’39] to model the heating value from proximate analysis values [39] and to predict the elemental composition from proximate analysis.[40] The data on the pH and mineral elements analyses of CS-derived biochars are also listed in Table 2. The pH increased with temperature resulting in biochars that are more alkaline in nature probably due to the concomitant decrease in acidic functional groups (section 4.3) and increase in ash content.[16] Similar patterns are observed when comparing the findings of this study with literature (Fig 3).

The best fit was obtained with a polynomial equation of second order resulting in a correlation coefficient of 0.597. The correlation coefficient drastically improves to 0.903 if the data point from Mullen et al.[28] is removed. The reason(s) for the comparatively low pH value of the CS biochar produced at 500°C are not clear. All authors but one [32] used either distilled or deionized water for the determination of pH, however the w/v ratio of char to water ranged from 1: 5to 1: 100. [28,33] The ash content of Calvelo Pereira *et al*. (2014) 550°C biochar was 10.8% while Mullen’s (2010) 500°C biochar contained 32.8% ash.[28,37] The implication that a high ash content biochar gives rise to a low pH is contrary to the trends reported.[41,16] Calvelo Pereira used a TGA to heat the sample under N_2_ atmosphere to 900°C before oxidizing the specimen.[37] As indicated by Enders et al. such high temperature could have caused the loss of various mineral constituents such as P, K, S and carbonates and result in the apparent low ash value.[16] It is therefore conceivable that the actual ash content of both Mullen’s and Calvelo Pereira’s biochars was more similar than reported, and hence not the primary reason for the observed difference in pH. It is worth noting that, in contrast to others, Calvelo Pereira et al. heated the biochar—water suspension in a water bath to about 90°C, stirred for 20 min and subsequently cooled down the suspension to room temperature for pH measurement in accordance with Ahmedna et al. method for pH determination.[41] Such heat treatment may drive out dissolved CO_2_ and other volatile acids thus increasing the pH. It is suggested for the biochar research community to either agree on an existing standard or practice for the determination of pH, or develop a new standard that harmonises the biochar: water ratio and minimizes interferences from dissolved CO_2_. In addition, a more accurate predictor of biochar’s impact on soil pH would be the determination of its acid neutralizing capacity (% CaCO3 equivalent).[42]

The elemental composition of the biochar is presented in Table 2. The carbon content in the biochar increased from 45.5% to 64.5% with increasing pyrolysis temperature, whereas the oxygen content decreased corresponding to an increase in the carbon content. This finding shows that carbonization was promoted with increasing pyrolysis temperature.[43–44] Hydrogen and oxygen losses at high pyrolysis temperature were due to the cleavage and breakage of weak bonds within the biochar structure. [36]

**Fig 4:**
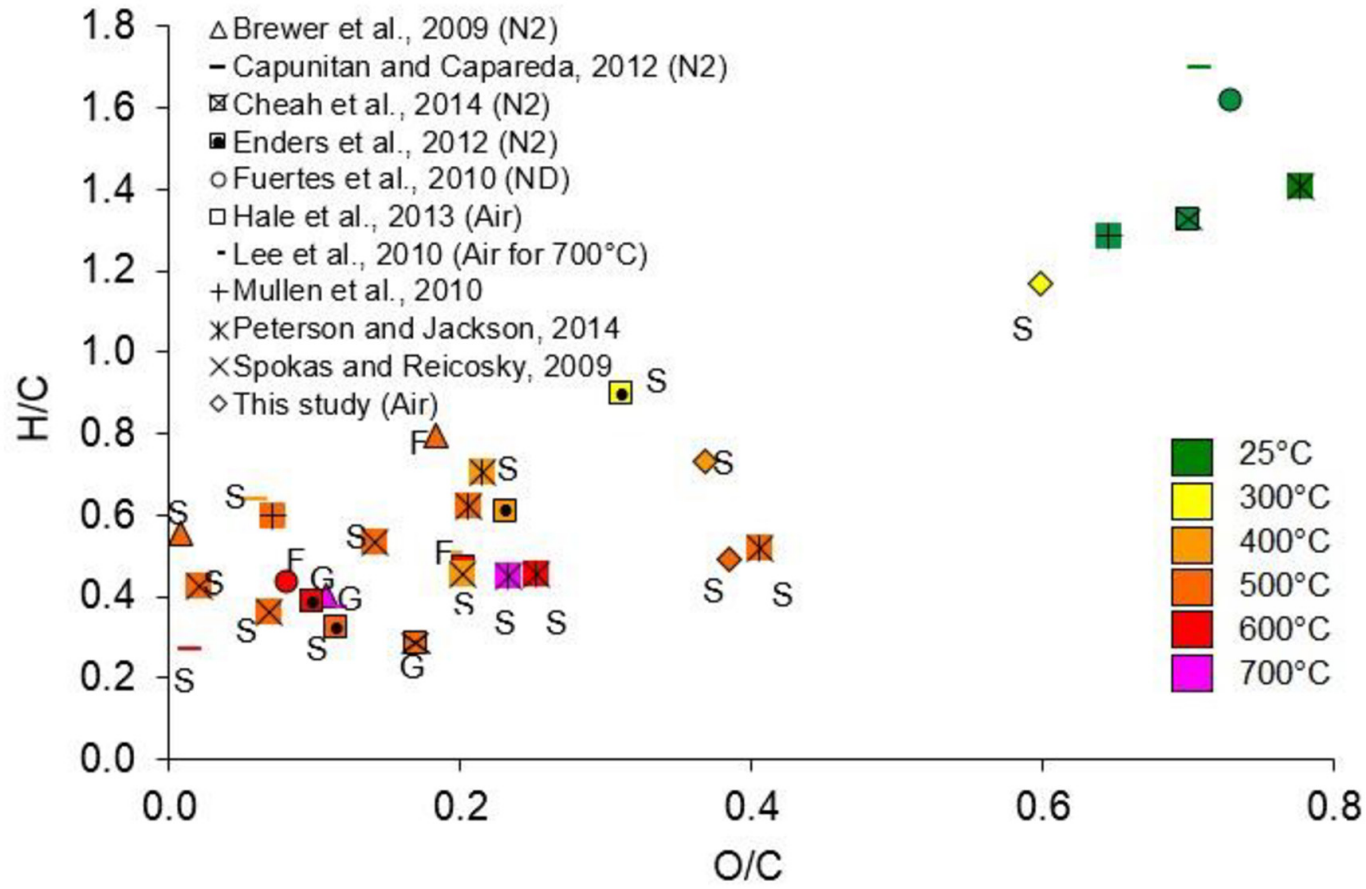
Van Krevelen diagram of original and carbonized corn stover. (S–slow pyrolysis; F–fast pyrolysis; G–gasification; ND–not determined). (n = 31).

The total N content for the experimental chars at 300°C, 400°C and 500°C were found to be 0.63%, 0.42% and 0.25%, respectively (Table 2). The total N content declined with increased pyrolysis temperature–suggesting an ignition loss of N during pyrolysis of corn stover. Yuan et al investigated the effect of pyrolysis temperature on physico-chemical properties of corn straw and observed similar patterns.[45] A van Krevelen plot summarizing original and carbonized corn stover produced by various research groups worldwide under different process conditions is shown in Fig 4.[16,17,18,25,26,27,28,34,46,47] Overall the hydrogen and oxygen content decreases with increasing temperature suggesting a greater hydrophobicity of the biochars, which agrees with the formation of more aromatic compounds as was evidenced by the FTIR analysis of the biochars (section 4.3). Variability in molar H/C and O/C ratios of original and carbonized corn stover at a given temperature are probably due to the presence of mud and other foreign substances in some cases, different holding times used, and uncertainties associated with representative sampling and the elemental analysis itself.

Variability in molar H/C and O/C ratios of original and carbonized corn stover at a given temperature are probably due to the presence of mud and other foreign substances in some cases, different holding times used, and uncertainties associated with representative sampling and the elemental analysis itself.

The total base cations and total P values of corn stover biochar were found to increase with pyrolysis temperature. The higher content of base cations and total P in the biochars are due to the concentration of relevant chemical elements in the biochars during the pyrolysis process [48]. The recycling of the soil nutrient is often desired to maintain soil fertility and improved crop productivity that can be achieved by the application of biochar to soil as biochars contain essential nutrient need to the soil. In addition to nutrient recycling, biochar has been proved to reduce chemical fertilizer application to soil.[49]

### 4.2. Surface area and pore properties of biochar

**Fig 5:**
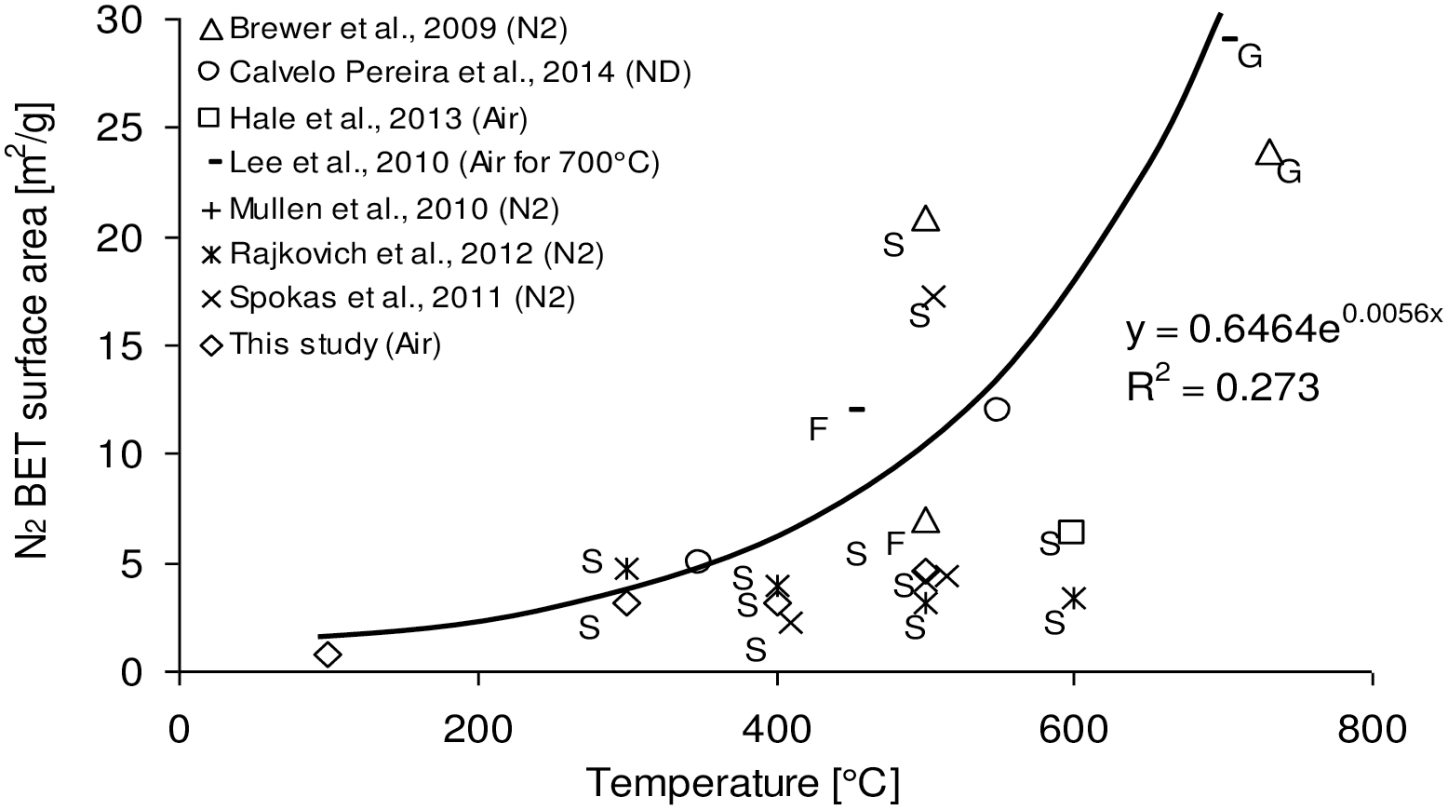
Effect of pyrolysis temperature on BET (N_2_) surface area of biochar produced from corn stover under slow (S) or fast (F) pyrolysis or gasification (G) conditions (n = 23).

Table 3 describes the surface area and pore properties of biochar. The BET surface area of the corn stover biochars increased with increasing temperature. This phenomenon can be generalized as shown in Fig 5. As pyrolysis temperature increases pore blocking substances are driven off or are thermally cracked thus increasing the externally accessible surface area. However, prolonged holding times can have the opposite effect since reactions continue at the pore surface area causing a decrease in micro pores and a shift towards meso- and macro pores. The relatively low surface area observed for corn stover biochar is probably due to the inorganic material that partially fills or blocks the micro pores.[50,38]

**Table 3:** Surface area and pore properties of corn stover biochars.

Biochar sample	S_BET_^1^ (m^2^g^-1^)	S_micr_^2^ (m^2^g^-1^)	Vt^3^cm^3^ g^-1^	V_micro_^4^ (cm^3^ g^-1^)	D_ave_^5^ (nm)
300°C	3.19	-	0.00843	Not detectable	10.6
400°C	3.17	0.127	0.00689	Not detectable	8.69
500°C	4.58	1.02	0.00722	0.000294	6.30

^1.^ N_2_BET surface area.

^2.^ Micro porous surface area by t-plot method.

^3.^ Total pore volume by from single point adsorption at relative pressure close to 0.995.

^4.^ Micro porous pore volume by the t-plot method.

^5.^ Average pore width, estimated by 4Vt/S_BET_

The reported surface areas may temporarily increase if biochars are added to soil and pore water leaches out minerals with low affinity for the char surface. For example, Hale *et al*. determined the BET (N_2_) surface area of unwashed and leached corn cob biochar and noted an increase from 36.4 to 84.3 m^2^/g.[51] It is reasonable to expect that humic substances and other organic matter, heavy metals, micro-organisms and tertiary roots may occupy eventually the vacated pore volumes. An exponential equation was the best fit for the data presented in Fig 5. However, the correlation coefficient of 0.273 suggests that other process parameters than temperature has a more pronounced effect on BET surface area. Most biochars were produced under slow pyrolysis conditions while the BET surface area of the two fast pyrolysis biochars from bubbling fluidized bed reactors are nested within the slow pyrolysis biochars. Based on Antal et al.’s experience the accidental exposure of carbon to air during carbonization dramatically affects its surface area and pore properties.[50] There appears to be a tendency in Fig 4 that the presence of air results in corn stover biochar with lower BET surface area. However, more data are required to confirm this apparent trend. The BET surface area may also be affected by variations in sample preparation (crushing and dry or wet sieving), the degassing method (vacuum vs flow), degassing temperature and duration, quantity of sample used during surface area analysis, number of replicates, using a representative sample quantity especially from upscaled reactors, as well as the presence of foreign substances (e.g. sand or soil) on the corn stover biomass during pyrolysis. Considering potential applications, the wide range of BET among the corn stover biochars may affect nutrient plant availabilities of co-applied fertilizers.[33]

### 4.3. FTIR analysis

**Fig 6:**
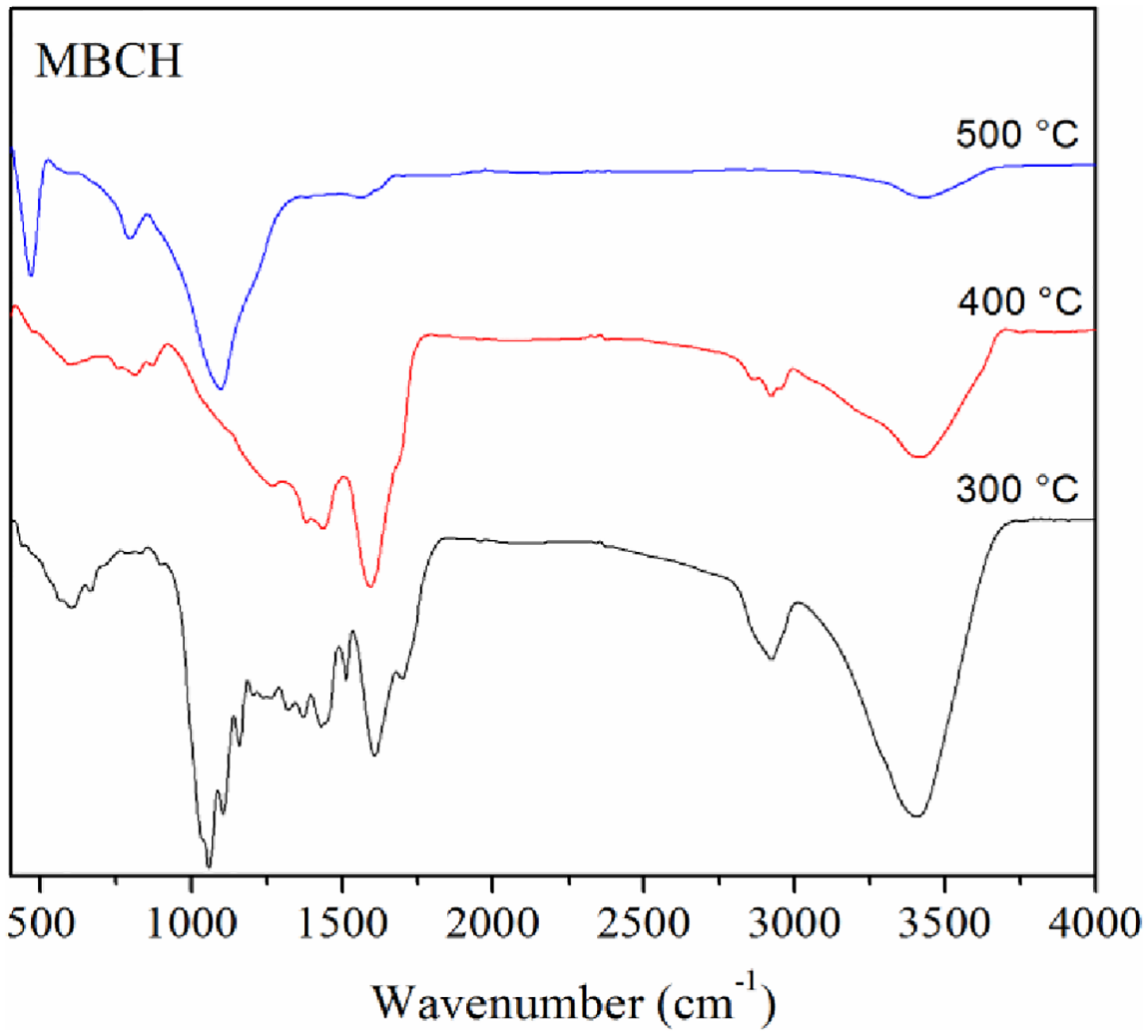
FTIR spectra of corn stover biochars produced at 300, 400 and 500°C.

The infrared spectra of the biochars are shown in Fig 6. The O–H stretch peak around 3600–3200 cm^-1^ is clearly visible in the char produced at 300°C but decreases as pyrolysis temperature increased representing dehydration of cellulose and ligneous compounds.[52–53] The presence of uncharred biomass in corn stover char produced at 300 and 400°C is exemplified by CH_2_ peaks at ~2900 and ~1400 cm^-1^.[54] Peaks of aliphatic C-O-C (1046 cm^-1^) and alcohol-OH (1160 cm^-1^) groups indicate the presence of hemicellulose and cellulose in corn stover biochar (300°C) suggesting the incomplete charring of corn stover at this temperature.[52,54] This also agrees with XRD and TGA results discussed further in sections 4.4. and 4.5. Corn stover biochar produced at 300°C is therefore expected to be at least partially biodegraded in soil.

### 4.4. XRD

**Fig 7:**
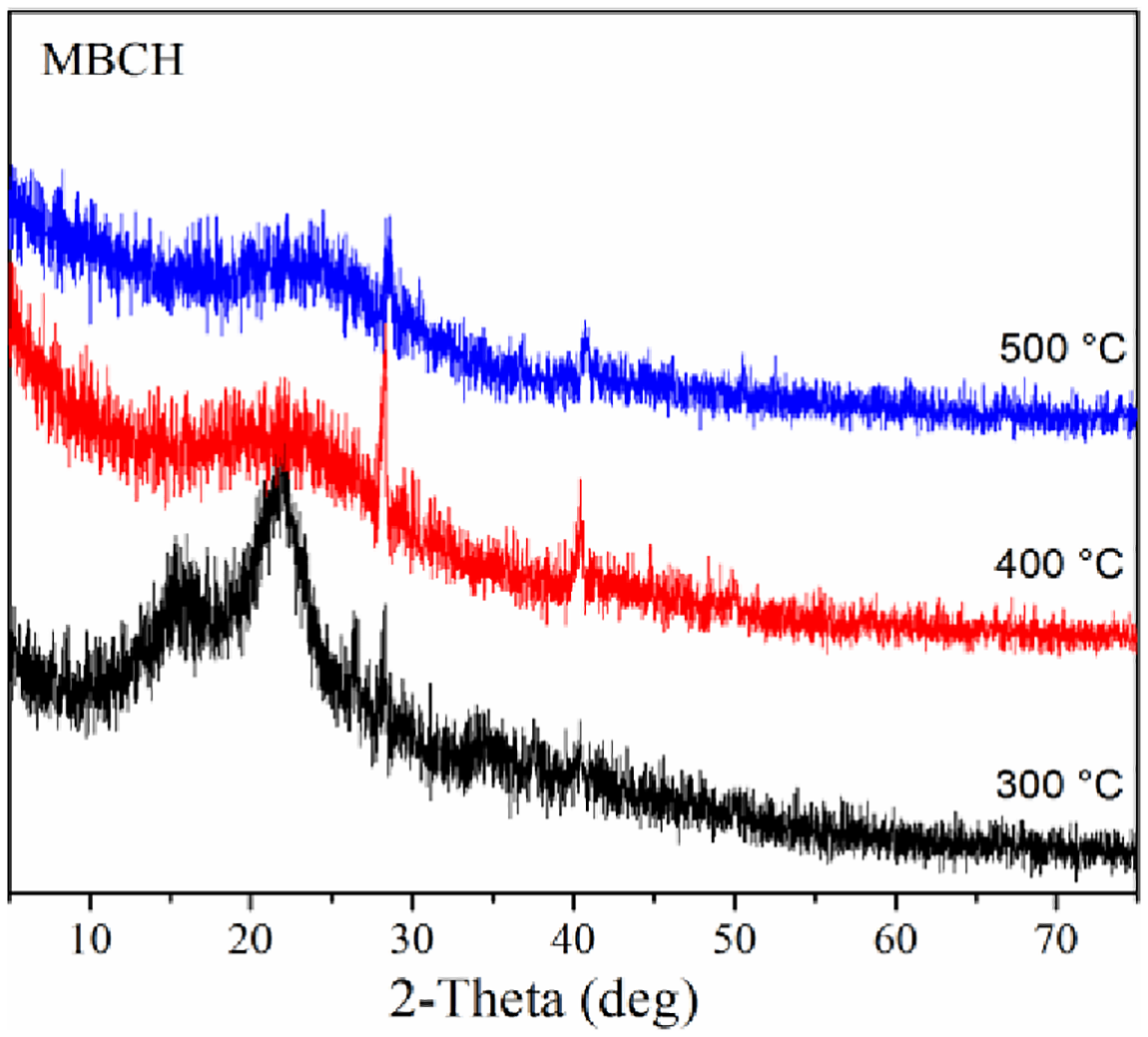
XRD spectra of corn stover biochars produced at 300, 400 and 500°C.

X-ray diffraction is a valuable approach to investigate the biomass crystallinity and biochar structure. [55] XRD results for corn stover biochars are illustrated in Fig 7. Two narrow, sharp peaks at the 2θ values around 16° and 22° for the control were assigned to the crystalline region of cellulose in wood (Yang et al., 2007).[56] For biochar-300°C, these two peaks decreased in intensity and became broader, indicating that some partial crystalline structure of cellulose was lost.[53] Although the biomass was partially decomposed at 300°C, the crystalline structure of cellulose remained due to the thermal stability of cellulose at such low temperatures. However, these two peaks were hardly visible in the XRD spectra for Biochar-400°C and Biochar-500°C indicating that crystalline cellulose was destroyed during char formation at 400°C and above.

### 4.5. Thermogravimetric analysis of corn stover biochar

**Fig 8:**
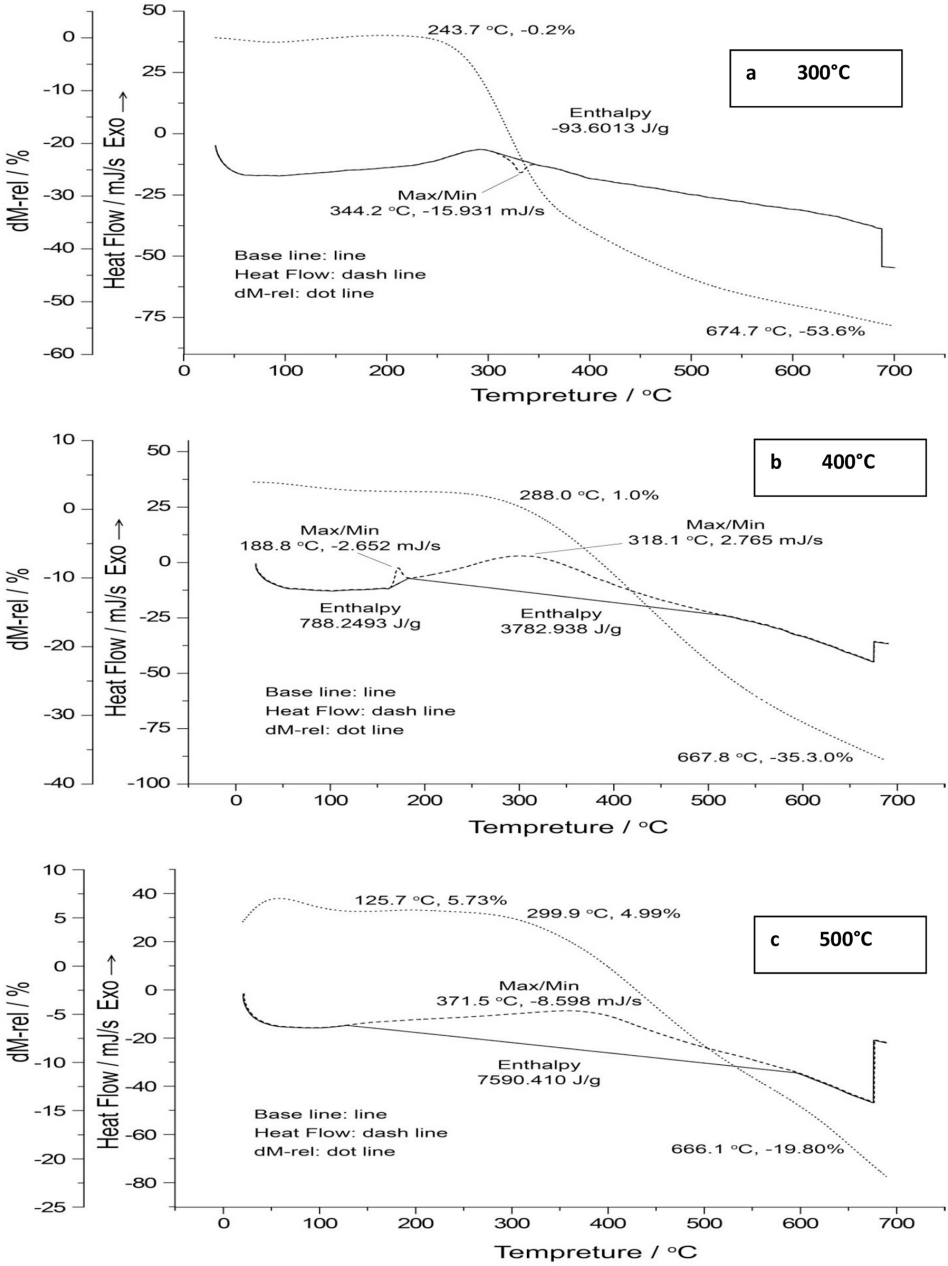
TGA/DTG spectra of corn stover biochars produced at, 300°C, 400°C 500°C obtained in a N_2_ atmosphere.

TGA and derivative thermogravimetric (DTG) curves are shown in (Fig 8a, 8b and 8c). TGA of experimental biochars was carried out to study their pyrolytic performance and thermal resistance. The TGA weight loss at temperatures upto 130°C was due to moisture loss. The subsequent weight loss of corn stover biochar (300°C) at 244°C and above may be attributed to thermal degradation of uncharred hemicellulose residues followed by uncharred cellulose and lignin. The onset temperature for TGA weight loss of biochars produced at 400 and 500°C shifted to 288 and 300°C, respectively, suggesting the presence of uncharred cellulose and lignin, which are more heat-stable than hemicellulose.[57] However, XRD analysis showed that crystalline cellulose disappeared in corn stover biochars produced at 400°C and above suggesting that lignin or other volatile pyro-compounds were responsible for the observed weight loss.

The TGA curves also show that biochar produced at 500°C exhibited the greatest thermal stability with an overall weight loss of 20% after heating to700°C,followed by chars produced at 400°C (35%) and 300°C (53%). The results confirm findings of Kim et al. that with increased pyrolysis temperature, biochars from pitch pine woodchips become more stable.[58]

### 4.6. CP/MAS ^13^C NMR

**Fig 9:**
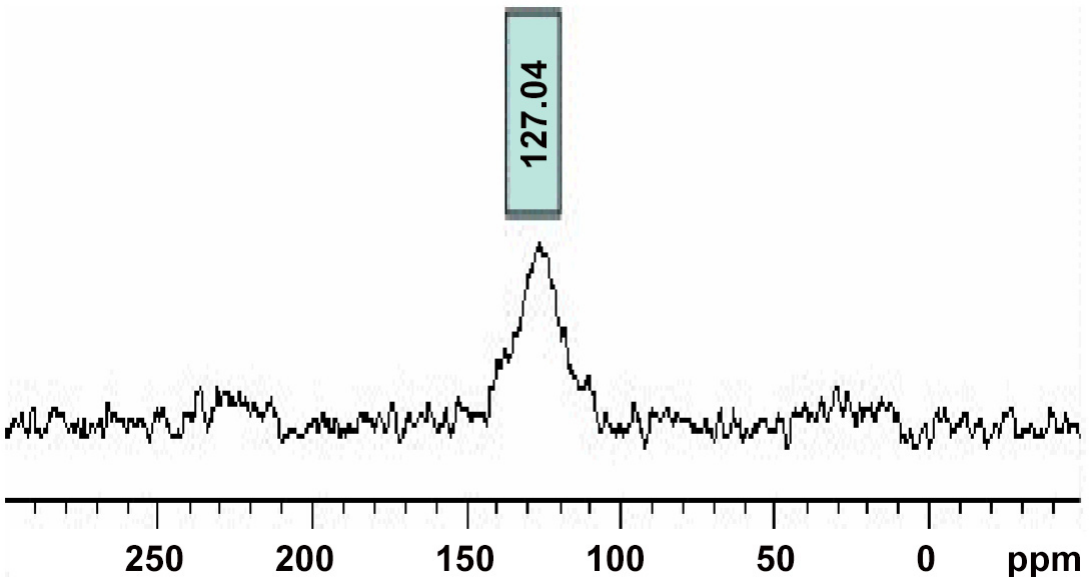
^13^ C NMR Spectra of Biochar- 500°C.

In order to evaluate the aromaticity of corn stover biochar produced at 500°C, CP/MAS ^13^C NMR was deployed. A dominant broad peak (Fig 9), at around 127 ppm was observed which can be attributed to aromatic carbon resonance. [58] The subordinate small and broad peaks around 205 ppm and 45 ppm for Biochar-500°C are due to spinning side bands originating from the dominant aryl peak.[59] Peaks at 50–100 ppm indicative of carbohydrates, were absent thus confirming that the observed weight loss in Fig 8 was not due to hemicellulose or cellulose.

### 4.7. Net energy balance for pyrolysis

The higher heating values (HHV) and lower heating values (LHV) of corn stover feedstock and corn stover biochars have been given in Table 4. The results show that corn stover biochar has significantly greater LHVs than corn feed stock on dry basis. However, gross and net energy value of biochars prepared at 400°C and 500°C did not differ significantly. While net energy balance is higher at higher temperature. Previous studies suggest that pyrolysis technology has potential to produce more surplus energy in addition to biochar for land applications is more attractive being environment friendly. [60]

**Table 4:** HHV and LHV of corn stover and biochars produced at 400 and 500°C.

Material	HHV MJ/Kg	LHV MJ/Kg	H%	Moisture%	Maximum energy from pyrolysis MJ/ kg
Corn Stover	18.18±0.06 a	16.74±0.06 a	5.89±0.02 a	5.4±0.2 a	-
Biochar (400°C)	27.50±0.08 b	26.60 ±0.08 b	3.9±0.3 b	1.65±0.09 b	8.03
Biochar (500°C)	27.6 ±0.2 b	27.0 ±0.2 b	2.7±0.1 c	1.18±0.04 c	10.12

Mean values followed by different letters within the same column are significantly different at P<0.05. Where H% = Hydrogen Percent

### 4.8. Biochar Characteristics and Carbon Stability

Table 2 shows that with increasing temperature there is an increasing trend in carbon content of biochars. Lowest volatile matter content (33.8%) was recorded in the CS char produced at 500°C and H/C value of 0.493 lowest among the CS chars indicated that chars produced at 500°C have highest stable carbon among the experimental biochar. These findings are very close to the Enders et., al.[16] about carbon stability of the chars. FTIR,TGA and ^13^C NMR confirmed the presence of more aromatic stable carbon in biochar produced at 500°C. Thus pyrolysis at 500°C may be a potential approach using crop residue for producing biochar for carbon cut off and green energy.

## 5. Conclusion

The biochar yield decreased when the pyrolysis temperature increased. The physico-chemical and structural characteristics of biochar were significantly influenced by pyrolysis temperature. The degree of carbonization for biochar was accelerated with increasing pyrolysis temperature from 300°C, 400°C to 500°C. TGA indicated that increased pyrolysis temperature developed more stable form of biochar. As the pyrolysis progressed, oxygen and hydrogen were removed, leaving to form more aromatic carbon bonds. The results of ^13^C NMR confirmed aromatic carbon in corn stover biochar at 500°C. Result suggested that corn stover pyrolysis is pertinent to reduce carbon emissions with net positive bioenergy production and its use as soil amendment. Comparison of findings with literature showed that biochar production from corn stover is influenced by various factors. Significant non-linear correlations between pyrolysis temperature and yield and pH were noted. Contributing factors for poor correlations between pyrolysis temperature, ash content and BET surface area include the presence of dirt from the field which can artificially increase ash content and block pores but also affect biochar yield and pH. Moreover, it was found that most studies appeared to analyse samples with one replicate only using various standard and non-standard methods. Issues of representative sampling and temperature measurements, especially in pilot- and industrial-scale reactors further complicate data comparison. It is suggested for the biochar community to establish an international committee to develop and agree upon standardized sample preparation and analysis methods to facilitate data interpretation and reliability.
